# Phantasmata
*"Phantasmata; or, Illusions and Fanaticisms of Protean Forms productive of Great Evils." 2 Vols. 8vo. 1857. By R. R. Madden, F.R.C.S., Eng., M.R.I.A., &c.


**Published:** 1858-01-01

**Authors:** 


### Art. VIII.—PHANTASMATA.*

It is one of the elementary laws of matter, that, when in motion, it has the power of communicating to other matter a similar motion to its own. This is not only true of masses dynamically considered, but also of molecular and chemical conditions, in which changes in progress have the power of inducing similar changes in substances similarly constituted; as in the case of fermentation. The dynamics of mind have been less investigated than those of matter; yet every step taken in this course shows more and more clearly that mind individual and mind collective, both act in accordance with certain definite laws, not dissimilar in nature and general results to those of matter. One of the most interesting results obtained from such inquiries, is the more clear comprehension of certain *class* states of mind—states produced in small or large communities as if by a veritable fermentation—a propagation of impulse molecularly amongst the masses, producing true epidemics of thought, emotion, or volition, as defined in their symptoms, as obscure in their source, as erratic and uncertain in their progress, often as deplorable in their results, as epidemics of cholera, plague, or small-pox.

Scientific psychology is a study of late invention; still imperfect in many of its details; not at all reduced to the condition of an exact science;—very imperfect still is our knowledge especially of epidemic crime and insanity; of epidemic emotion and volition. Yet the subject has of late years attracted much attention, and many most valuable contributions to its literature have been received. The most recent and most compendious treatise which we have seen, is that which forms the especial object of this notice. Mr. Madden has been well known, both to the profession and to the literary world generally, for about a quarter of a century, as an able and popular writer. His work on the "Infirmities of Genius," of which we have made some use in another part of this Journal, has for many years been a standard favourite. The present one is of a more weighty and learned cast, as becomes the more serious and important subject. It is full of matter, well-arranged, well-digested, and admirably calculated to illustrate the deeply-interesting subject under treatment. As to the precise object of the work, we will let Mr. Madden speak for himself, by which we shall see the very comprehensive views taken of the bearings which these studies have upon social and political science:—

* "Phantasmata; or, Illusions and Fanaticisms of Protean Forms productive of Great Evils." 2 Vols. 8vo. 1857. By R. R. Madden, F.R.C.S., Eng., M.R.I.A., &c.

"The subject of this work has largely to do with the failings, and infirmities, and passions of mankind, and their accompanying disorders of the imagination; for to these sources must we attribute the epidemic fanaticisms which we meet with in history *and elsewhere*—simulating at one time an ardent zeal for religion, at another a glowing love of liberty; now a laudable ambition to rise in the world, to attain to power, to obtain wealth, to add field to field, possession to possession, dominion to dominion; anon a strong wish and settled purpose to dominate over others, to master their wills, to invade their rights, to trample down their inferior intelligence, weaker powers, or feebler energies of mind or body.

"We are accustomed to regard passing events of an extraordinary character which disturb society as indications of rather too much political excitement, or polemical heat, sectarian strife, competition in trade, monopoly in patronage and preferments, an insufficient police force, an inadequate representation, too little rationalism in religion, or reverence for law, or devotion to material interests, or knowledge of the true principles of political economy. We find it saves the trouble of thinking to fall into this way of viewing remarkable outbreaks of popular frenzy, like those of the Reign of Terror of the French Revolution, in 1792 and 1793; outbreaks of intolerance and immanity in Spain and Portugal in the times of the Inquisition; outbreaks of barbarity in England and Scotland and the New England States of America, in the proceedings against witches; outbreaks of superstition in various countries in regard to new revelations of pseudo-saints, pseudo-'spiritualists,' pseudo-seers of mesmerism claiming prophetic gifts; outbreaks of a raging avidity for sudden gain—for means, no matter how they may be acquired, to live luxuriously, or to seem to others to be rich. . . . .

"The madness of the various forms of fanaticism is not confined to individuals—it extends to communities, at times and intervals more or less widely separated, and seizes on the minds of nations at periods of greater intervening distances, that have been terminated by great wars, or other grievous public calamities. . . . . Such fanaticisms have all the distinguishing characteristics of epidemic mental disorders."—Preface, p. 6-8.

Mr. Madden attributes these fanaticisms to an epidemic disorder of the moral sense, which is not to be removed "or remedied by materialism professing Christianity, or sanctimony and sectarianism proffering for genuine religious instruction the teachings of strife and bitterness." He notices also, as a remarkable fact, that the most extensive fanaticisms that the world has seen have not originated with the poor and uneducated, but with persons of abilities and education; with, in fact, the "educated men." One remark on this subject is impressive and important:—

"It is said that 'man, ignorant and uncivilized, is a ferocious, sensual, and superstitious savage.' But there may be a great deal of savagery in the heart's core of civilization, when the intellectual faculties alone have been educated, and the moral feelings and affections left untaught. To use common but significant terms, you must educate the heart as well as the head."

The first illustrations of epidemic insanity which are given, are derived from the various disorders and commotions which affected various parts of Europe in the fourteenth, fifteenth, sixteenth, and seventeenth centuries, and "signally in the convents of several of the religious orders in France, Spain, and Germany." These disorders appear to be both contagious and epidemic. With many of the facts we are sufficiently familiar, through the medium of Hecker's valuable treatise on the "Epidemics of the Middle Ages." Mr. Madden makes some valuable observations, from which we shall quote:—

"The mind and body reciprocally and mysteriously affect each other. To determine where disorders of the body cease to be mere physical derangements, and where mental maladies supervene on bodily ailments, and to distinguish between states of health in which bodily functions or organs are affected, and mental faculties or moral feelings are perverted, requires a large amount of knowledge of medical philosophy, as well as of practical acquaintance with medical pursuits."

He enlarges upon the necessity for calling in history to aid us in our researches, and "it might be added, we must have that historic light before we can understand how many degrees there are of enthusiasm, and of excitability of the nervous system, which amount not to the temperature of the mind at which reason ceases to be recognised as a controlling power."

Mr. Madden holds that the epidemic forms of mental excitement are not merely matters of history, but are liable to recur at any times and seasons when the proper conditions are present; perhaps in modified forms, in accordance with the changed aspects of society, of science, and of belief; but still, that imagination will always, to the end of time, and in all forms of civilization, have a tendency to exert a powerful influence over "the production of disease, and morbid sentiments that border on insanity."

That we are not free from these tendencies is a position capable of ample illustration; and Mr. Madden, after alluding to Mr. Thoms of Canterbury, and Johanna Southcote, thus proceeds:—

"Has the enlightenment of the nineteenth century so entirely dissipated the dark, thick mists of demented superstition that no traces of it are to be found in modern English and American records? In what language is the future word-painter of Welsh history to depict the strange antics and the frantic orgies of the Jumpers and Revivalist fanatics?

"Will Macaulay 'come down' to the period of the field-meeting of the saints, and the love-feasts of the brethren and sisterhood of the elect in Wales? Or will Alison 'finish Europe' with a chapter on modern miracles, furnishing a *résumé* of the phenomena, and an elucidation of the mysteries of clairvoyance?

"Shall we read in that chapter, of revelations from the other world by persons in the 'superior condition,' solemnly announced in the presence of Christian ministers, of dignitaries of the Church, impugning doctrines of Christianity which are deemed fundamental truths in all its churches?

"Must we go back to the middle ages for sorcery and dealings with 'Satan's invisible kingdom?' Or may we not only have to cross the Atlantic on a voyage of discovery for devils, and those who commune with 'fallen angels' and 'inferior spirits?' Have we not in America, at the *séances* of the spirit-rappers, scenes which may remind us of the 'sabbaths' of assembled witches; media stationed in circles, intent on conjurations; discoursing in a jargon scarcely intelligible to the uninitiated; invoking spirits—some 'disobedient,' 'mischievous,' 'perverse,' 'mocking,' and 'mendacious;'—others 'benign,' 'angelic,' and 'divinely gifted intelligences?'"

After noticing the Mormons, and various other deluded associations, we find the following shrewd observations:—

"We have abundant confessions of compacts with devils, preparation of philtres, all kinds of extravagant practices of a sortilegious kind, in the records of judicial proceedings of the middle ages, and of those occurrences which took place under the sanction and auspices of spiritual superstitions, carried into effect by the strong hand of the secular arm at the gibbet and the stake.

"And in the nineteenth century we have no dearth of avowals of sorcery, of interviews with Satan, of power derived from his angels, of the perpetration of fearful crimes committed by Satanic suggestion; but not in the same places and under the same circumstances as in the middle ages. *We have them now in lunatic asylums*, on the part of persons who are restrained on account of their insanity, and not burned in the market-places, on the plea of vindicating God's honour." —(p. 11.)

After noticing the "fanaticism of infidelity," and its effect in producing the horrors of the French Revolution, Mr. Madden observes—

"An infidel periodical literature in England is accomplishing a similar mission there—slowly, perhaps, but surely—among the great masses of the people; and where its propagandism of infidelity, and the vulgar cynicism of socialism, does not extend, corresponding results may be expected from the prevalent indifference in matters of religion which characterizes the literature, science, and philosophy of our time, which pervades our journalism, and lurks under the folds of the drapery of fashion, as well as in the precincts of St. Stephen's Chapel."

After these illustrations of our author's style of thought, we propose to notice very briefly a few of the interesting topics on which he touches. A short notice of the life of Swedenborg is given, from which it would appear that he was the subject of almost constant hallucinations both of the eye and ear, as we feel bound to reject entirely the theory of imposture. He was accustomed to hold frequent and long conversations both with angels and men deceased. He talked with St. Paul during an entire year ; with St. John, Moses, and Luther. He says, " Lest any one should call this an illusion, or imaginary perception, it is to be understood that I am accustomed to see them when perfectly wide awake, and in full exercise of my powers of observation." The reason why the speech of an angel is not heard by the bystanders is, that it " finds entrance into a man's thoughts, and reaches his organs of hearing from within." This, or some analogous form of expression, is very common amongst those who suffer from auditory illusions. Thus the prioress of the convent of Soudum, tormented by the demon Behemoth, heard him speak "par une locution qu'il un faisait dans ma tête."

On the subject of the well-known visions and revelations of St. Teresa, Mr. Madden says :—

" It is impossible for a medical man to read this account (an account of her bodily sufferings before given)—of the occasional falling into a lethargic state, fits of fainting and swooning, violent spasms and pain at the heart, temporary loss of reason, shrinking of the sinews, oppression, with a profound sense of sadness, biting of the tongue in many places when out of her senses, inability to swallow any liquid, distortion of the whole frame as if all her bones were disjointed, subsequent inability to stir hand or foot for some time, and a generally-diffused soreness, so as to be unable to bear being touched—without coming to the conclusion that the sufferer laboured under physical disease of a low nervous or gastric kind, with continuous fever, probably complicated with epileptic tendencies."

Elaborate and interesting notices are given of various forms of epidemic manias ; amongst others, of Witchcraft, of Lycanthropy, Epidemic Hysteria, Convulsive Chorea, Manie de la Danse amongst the French, the Tarantula dancing-mania of Apulia, the Flagellation mania, and the Migratory Epidemic. The last is thus noticed :—

" At various times in the middle ages, the minds of a multitude of people seem simultaneously to have been affected with this universal feeling of *malaise*, accompanied by an irresistible and unaccountable impulse to go forth and walk out of one's own land and place in society, to move with masses of people with some apparent instinct of a high purpose ; at one period they appear, as it were, on a pilgrimage, but without any definite object, or fixed shrine—like the Bianchi in the thirteenth century, wandering *en masse* from one end of Italy to the other, making no proclamation of plan or object, but moving onward with a dim, confused idea, that God's honour was in some way or other to be promoted by these peregrinations."

These migrations are to be distinguished from the Crusades, which were epidemics of another class.

About half of Mr. Madden's second volume is occupied with a carefully-prepared history of the life and character of the Maid of Orleans, Jeanne d'Arc, founded on the work of M. Quicherat, whose researches were undertaken for the Société de l'Histoire de France. The result arrived at appears to be, that Joan of Arc was affected to some extent with thermania—that she was subject from her earliest infancy to illusions and hallucinations of sight and hearing—that the intensity of her enthusiasm projected outwards the promptings of her own mind, so as to give them substantive existence, and that they were acted upon as inspirations. It appears fully proved, however, that her life and morals were perfectly pure and irreproachable, and that her execution was nothing more nor less than judicial murder. Mr. Madden does not reject the idea that she had in some sort a divine mission to perform, as would appear from the concluding sentence of his chapter on her inspiration :—

"Agents of this sort have been used by Divine Providence for great purposes in all times, and in many regions of our globe. . . . . With the several Scripture records of inspirations given to women for the accomplishment of great and good designs, the history and mystery of the visions of Jeanne d'Arc may possibly be found to be in nowise inconsistent."—(Vol. ii., p. 226.)

On the subject of epidemic mania in convents, much useful information is afforded in these volumes, showing how, under mental influence, the most extraordinary phenomena were manifested in frail, weak bodies, and how singularly these phenomena coincided in the various places in which they appeared ; also, how very similar they are to the results claimed to be produced by modern mesmerism, such as magnetic sleep, insensibility to pain, clairvoyance, influence over volition, &c. &c. But we must close our notice and extracts, strongly recommending these volumes to the attention of all who feel an interest in the very obscure yet important subjects on which they treat.

